# Meaningful Work and Satisfaction with Life: A Case Study from a Supported Employment Program—Colombia

**DOI:** 10.3390/bs12070229

**Published:** 2022-07-13

**Authors:** Merlin Patricia Grueso Hinestroza, Concha Antón, Mónica López-Santamaría

**Affiliations:** 1 School of Management and Business, Universidad del Rosario, Bogotá 111711, Colombia; monica.lopezs@urosario.edu.co; 2 Department of Social Psychology and Anthropology, Faculty of Psychology, University of Salamanca, 37005 Salamanca, Spain

**Keywords:** meaningful work, work experiences, satisfaction with life, Colombia, armed conflict

## Abstract

Work helps to satisfy instrumental and transcendental needs. For people affected by armed conflict, work has an additional value because it helps them overcome the social disadvantage they suffer; however, topics such as meaningful work—MW—have been poorly studied in this type of population. Based on the above, we propose to analyze the relationship between meaningful work and satisfaction with life in one of the largest private supported employment programs for people involved in the armed conflict in Colombia. To this end, a nonexperimental, quantitative case study was conducted with 62 employees of that employment program. To collect the data, a survey with two measurement scales was administered: *Work as Meaning Inventory* and *Satisfaction with Life Scale*. Sociodemographic variables were also obtained. The results demonstrate that meaningful work has a significant effect on satisfaction with life (R^2^ = 0.28, *p* < 0.00). We conclude that having meaningful work that provides a sense of belonging, interpersonal connection, and attachment generates greater satisfaction with life in the workers involved in the analyzed program. We also discuss the implications of this research for companies and public policy in Colombia.

## 1. Introduction

Work helps to satisfy instrumental and transcendental needs; it is a key dimension in which people search for meaning [1]. The human need for meaningful work is well known [1,2]; indeed the current employment market conditions lead individuals to reflect on the meaning of, and reason for their actions, transcending the instrumental, technical, and operational dynamics of labor tasks [3]; thus, studying the processes of meaning has received greater attention in the work environment [1,4,5,6].

The concept of meaning is directly linked to human beings’ existence, and, in this sense, work is understood as a fundamental element of life [6,7]. The processes of meaning in work have been implicitly present in academic fields, including psychology, political theory, theology, philosophy, human resource management, and management and organization studies [5], and, even more, when this meaning is associated with actions that add value to society [6,8].

Several researchers highlight the importance of conducting studies that contribute to understanding work perceived as meaningful [6,7,8,9,10,11,12], especially in these turbulent times in which a constant transformation of work is taking place [12].

Everyone who has a job does not necessarily have access to meaningful work. Studies have shown that people who identify as sexual minorities, for example, systematically face barriers to decent and meaningful work, particularly when they are also restricted by barriers such as low socioeconomic status or marginalization [13]; despite these findings, little research has been done on meaningful work in socially disadvantaged populations.

The understanding of work experiences among people enduring socially disadvantaged conditions, such as the Syrian refugees [14,15,16,17] has increased; however, there are other populations in conditions of marginalization and social disadvantage that deserve attention from psychological research, such as people affected by long-lasting armed conflict. Thus, this study analyzes the relationship between meaningful work and satisfaction with life in a sample of people in a supported employment program aimed at people affected by the armed conflict in Colombia.

This paper first describes the context in which the research was conducted. Next, we develop the theoretical framework and empirical evidence that support the research question of the study. This is followed by sections on the methodological design, results, and discussion. Finally, the conclusions, limitations, and directions for future research are presented.

### 1.1. Context for the Study

The number of people trying to escape from wars or persecution is dramatically increasing [16,18]. In Colombia, armed conflict has been the main problem in the last six decades [19,20], which has been classified as one of the longest in world history. According the Single Registry of Victims managed by The Unit for the Attention and Integral Reparation of Victims of the Crime, by May 2022, 9,278,531 people were considered victims of the armed conflict [21]. In this context conflict, between 1958 and 2021, there were 373,629 fatalities: 97.04% were civilian victims [22].

With the purpose of promoting disengagement of the persons who have taken up arms and their reintegration into civil life, Colombia has faced 14 processes for 35 years and has managed nine programs regarding disarmament, demobilization, and reintegration for individuals and groups organized outside the law, individually and collectively [23]. According to The Agency for Reincorporation and Normalization in Colombia, by April 2022, 76,598 people from organized armed groups outside the law had been demobilized: 51,791 entered the process of reintegration, while 26,888 had already completed their process and hoped to effectively reintegrate into society [24].

In order to contribute to the end of the armed conflict and social restoration, and to favor the attainment of employment both for people in the process of reintegration and for those who have been recognized as victims of the armed conflict, the Colombian government has been developing labor inclusion models for these populations for several years, which, although they have yielded interesting results, still face great challenges, as described below.

#### 1.1.1. Employment Conditions of Ex-Combatants

Through the reintegration process, the ex-combatants acquire a civilian status, adapt to society, receive education, become employable or entrepreneurs, receive income for their work, and reestablish social ties [25]. The duration of Colombia’s reintegration program is approximately 6.5 years and involves eight dimensions: personal, productive, family, habitat, health, citizenship, education, and security [26].

The productive aspect oversees the promotion of income generation through employability or entrepreneurship. During the process of reintegration, achieving formal employment can be a complex task, largely due to social stigmas [27]. Despite the fact that there are several companies that hire people in the reintegration process, the unemployment rate of the participants in the reintegration process (18.2%) is higher than the national average (8.7%), as indicated by Guerrero [28].

#### 1.1.2. Employment Conditions of the Armed Conflict Victims

In Colombia, people who have been recognized as victims of the armed conflict have access to the Individual Integral Route. This comprehensive reparation to victims involves: monetary compensation or restitution of property, accompaniment for the effective enjoyment of rights in health, education, housing and employment, and income generation [29].

The academic research has proven that victims of conflicts are more likely to experience long-term unemployment because of the armed conflict [30,31]. The situation in Colombia reflects this trend [20,30,31,32,33], and the high unemployment rates of the victims of armed conflicts could be explained by their relatively insufficient credentials, low levels of education [25], and insufficient skills to participate in the urban labor market and networks [32], although there are also various business and tax incentives for companies that hire people who are victims of the armed conflict. 

In sum, people involved in the Colombian armed conflict (victims and demobilized) face similar employment challenges: higher unemployment rates [20,30,31,32,33] lower educational levels, as well as fewer skills and social contacts [25,32]. For this reason, there are public and private employment programs, such as the one described in this research, that develop labor inclusion models for the affected population.

## 2. Theoretical Framework

### 2.1. Meaningful Work

Meaningful work (MW) is a core construct that reflects its importance at the individual, organizational, and societal levels [34]; it has also been noted that it is crucial for organizations to engage and retain their employees [35,36] and is a topic highly valued by employees, even more than employment conditions such as income, job security, and promotions [37].

Meaningful work is defined as a “work experienced as particularly significant and holding positive meaning for an individual” [37] (p. 1). Rosso et al. [38] define meaningful work as providing workers with a positive direction. On the other hand, Allan et al. [39] define MW as “the global judgement that one’s work accomplishes significant, valuable, or worthwhile goals that are congruent work with one’s existential values” (p. 502). Finally, Steger et al. [6] point out that MW means a work significant and positive in valence, which means that the work is growth and purpose oriented rather than pleasure oriented. After a systematic review, Both-Nwabuwe [11] proposes a definition as follows “meaningful work is the subjective experience of existential significance resulting from the fit between the individual and work” (p. 7).

According to Allan et al. [38], those definitions correspond to two research streams that propose the understanding of the concept from a unidimensional perspective, e.g., [38] or from a multidimensional perspective [6,38].

As a construct, meaningful work has become increasingly relevant in the academic world [5,34,40,41]; consequently, a growing body of research has analyzed its relationship with individual and organizational outcomes [5,11], such as well-being [37,42], performance [37,40,43], satisfaction [6,34,40], and commitment to work [6,40]. In general, MW has a positive contribution to quality of life at work [8,9,11,44,45,46].

### 2.2. Satisfaction with Life

Satisfaction with life (SWL) is people’s assessments and feelings regarding their environment and themselves [47,48] According to Diener et al. [49], SWL is considered a subjective component of well-being and refers to a “cognitive, judgmental process” (p. 71). SWL can therefore be defined as a global cognitive judgment about life in which the evaluation is not based on objective parameters but rather depends on a comparison of life circumstances with one’s own internal patterns [50]. It has also been noted that life satisfaction affects people’s capacity for social connectedness, as well as their sense of self-efficacy, and has a close relationship with the fulfillment of life purposes [50].

### 2.3. Meaningful Work and Satisfaction with Life

Research evidence suggests that experiencing meaningful work is strongly associated with well-being outcomes, such as health [51,52], life meaning [51,52], and life satisfaction [6,39,51,52,53]. Additionally, Allan et al. [54] identified meaningful work as positively linked to a meaningful life and negatively linked to the search for meaning in life. In addition to the above, meaningful work has also been associated with lower levels of stress, anxiety [43], and depression [9,43,55].

Based on the theoretical assumptions and empirical evidence, and given the scarce research on meaningful work and its relationship with general life satisfaction in populations affected by armed conflict, the following research question (RQ) is proposed:

RQ: Meaningful work has a statistically significant and positive relationship with satisfaction with life among people participating in a supported employment program offered by a private company for people affected by the armed conflict in Colombia.

## 3. Method

### 3.1. Design

The study is a nonexperimental, quantitative theory-guided case study and uses a survey approach with a cross-sectional design.

### 3.2. Unit of Analysis

The study was conducted in one of the largest private supported employment programs that offers employment opportunities to people affected by the armed conflict, namely, people in the process of reintegration (i.e., former paramilitary and guerilla members) and victims. The program is 16 years old and is based on a supported employment model. The program analyzed operates in Medellín (Colombia) and is managed by a private enterprise that had received recognition because of the employment offer for the population affected by the armed conflict.

Supported employment programs are defined as “supporting people with disabilities and other disadvantaged groups to obtain and maintain paid employment in the open labor market” [56] (p. 9). Supported employment has promoted the employment of people with disabilities and, more recently, former drug addicts and prisoners [56]. This model has also promoted respect toward human beings, social integration, the empowerment of people, and dignity.

### 3.3. Inclusion and Exclusion Criteria

All workers participating in the employment program were eligible to participate in the investigation. No exclusion criteria were established for the sample.

### 3.4. Sampling Procedures

The participants were 64 individuals working in the employment program. After obtaining approval to conduct this study by the Ethics Committee from the University of Rosario, as well as from the employment program to disseminate the research packets to the participants, the employees were provided with a packet containing the informed consent, demographic forms, and research instruments. Data collection was completed in a quiet room, and each participant was tested individually. The participants required 30–40 min to complete their surveys. No participant complained about the number of items or the content. After a data revision, 62 surveys were used for the analysis because some participants did not fill out their forms correctly (*n* = 2).

### 3.5. Participant Characteristics

In this research, 55% of the sample were women and 45% were men; 42.2% were less than 30 years of age, 35.9% were between 30 and 39 years of age, and 21.9% were between 40 and 49 years of age. A total of 30% were victims of the armed conflict; 70% were in the reintegration process (i.e., former paramilitary or guerrilla members). The educational level of the participants was: 9.4% had completed primary school as their highest level of education; 46.9% completed high school; 34.4% completed technical education; and 9.4% completed the undergraduate level. Regarding marital status, 50% were single, 4.7% were married, 43.8% were common-law married, and 1.6% were divorced. Regarding the participants’ duration at the program, 31.3% had less than 1 year in the program; 43.8% had 1–3 years; 4.7% had 3–5 years; 4.7% had 5–7 years; 12.5% had 7–10 years; and 3.1% had greater than 10 years.

### 3.6. Measures

To develop the research, a survey with two measurement scales was used. The *Work as Meaning Inventory*(WAMI) [6] was used as the predictor variable, while the *Satisfaction with Life Scale* by Diener et al. [49], translated into Spanish by Atienza et al. [57], was used as the criterion variable. Sociodemographic variables were also collected.

*Work as Meaning Inventory* [6]. Some of the previous research used the WAMI, e.g., [6,58,59,60]; it is the most recommended measurement scale to examine relationships between the experience of meaningful work and certain antecedents or outcomes [11].

The WAMI is a measurement scale that comprises 10 items and assesses the degree to which people feel their work is meaningful. For the translation and adaptation of the scale into Spanish [61], guidelines were followed as in other Spanish studies (e.g., [58] performing a double translation: from the original to Spanish and from Spanish back to English). Translations were done by two independent professionals. To validate the correspondence of the items, we compared both English versions, the original and the translation from Spanish to English. Since an inconsistency was found with one of the items of the scale, it was necessary to eliminate one item, leaving nine items. A Likert scale with five response anchors was used; these anchors ranged from completely agree (5) to completely disagree (1). A higher score indicated having more meaningful work. Examples of the items in the WAMI include, “I have a good sense of what makes my job meaningful” and “My work helps me make sense of the world around me”.

To validate the factorial structure of the WAMI, a confirmatory analysis was performed based on a three-factor structure, as has been evidenced in previous studies [6,60]; however, the fit indices with a three-factor structure were not adequate (Table 1). Consequently, it was decided to test with a one-factor factorial structure, which obtains better fit indices, as occurred in the research conducted by Leonardo et al. [59] with the Brazilian population (Table 1). The reliability coefficient for the measurement scale was 0.954.

*Satisfaction with Life Scale*. For this research, the *Satisfaction with Life Scale* developed by Diener et al. [49] and translated into Spanish by Atienza et al. [57] was used. The general *Satisfaction with Life Scale* has five items that assess people’s overall judgment regarding life satisfaction. The questionnaire used a Likert scale with five response anchors that ranged from completely agree (5) to completely disagree (1). Thus, a higher score indicated greater satisfaction with life. Two examples of the items in the questionnaire are as follows: “In most aspects, my life is what I want it to be” and “Until now, I have gotten things from life that I consider important”. An exploratory factor analysis was conducted in which the result of Bartlett’s test of sphericity was 182.924 (*p* < 0.001), indicating that the five items were not independent. The Kaiser–Meyer–Olkin coefficient yielded a value of 0.82, and the total variance explained was 66.6% for the unifactorial model. The reliability coefficient for the elements classified was 0.86. These results were similar to those obtained by Atienza et al. [57].

### 3.7. Data Collection

The survey requested sociodemographic data without information that would allow identification of the participants, who are classified as a vulnerable population.

To administer the questionnaires, a time slot was established for two days in which the participants could answer the questionnaire with the presence of one of the researchers in the study. These surveys were administered in a space provided by the entity that administers the employment program.

### 3.8. Data Analysis

The data analysis was conducted with the SPSS statistical package. Confirmatory factor analysis and exploratory factor analysis were used to verify the factorial structure of the WAMI and *Satisfaction with Life Scale* respectively. Reliability coefficients were also computed for both scales. Descriptive statistics were calculated for the sociodemographic variables. For the research question test, a regression analysis was developed. To test the research question, we standardized the scores to eliminate sources of variance in the raw values [62]. Frazier et al. [63], for example, also indicated that, when standardizing scores, interpreting the effects of the predictor and or moderator variables is easier.

## 4. Results

### 4.1. Descriptive Statistics

First, we calculated the basic descriptive statistics for each of the variables used in the study. The results demonstrated that meaningful work obtained the highest average score, and its standard deviation was lower compared with satisfaction with life. Both variables scored greater than 4.0 (Table 2). Subsequently, we proceeded to calculate the correlations between the study variables. The results obtained show that the variables meaningful work and satisfaction with life are highly correlated R = 0.54, *p* < 0.01.

### 4.2. Testing the Research Question

To guarantee the validity of the regression model, we verified the degree of independence between residuals and demonstrated that the residuals are independent. Generally, the Durbin–Watson statistic fluctuates between 0 and 4, and the rule is that this value should be between 1.5 and 2.5. In this case, the Durbin–Watson statistic was 2.068; as a consequence, the residuals are deemed to be independent (Table 3). Similarly, the homoscedasticity was verified by observing no association pattern between the predictors and residuals. The verification of the assumption of normality showed that the residuals were distributed normally.

The effect size and power were calculated in G * Power, using: (a) Test family: *t* Test; (b) Statistical test: Linear Multiple Regression: Fixed Model, single regression coefficient; and (c) Type of Power analysis: Posthoc: Compute achieved power … Tails: Two. First, the effect size was obtained by calculating the explained variance (0.28) and the residual variance (0.72), resulting in an effect size of 0.388. An effect size of 0.30 indicates an effect that is large and potentially powerful in both the short and the long run.

Subsequently, the statistical power was calculated. The conventionally expected statistical power for an analysis is 80%, which means that there is a 20% probability of accepting the null hypothesis when it is actually false [64]. In our case, the use of a probabilistic error of 0.05, a sample of 62, and a predictor variable, obtains 0.99 of statistical power.

To test whether meaningful work significantly predicted participants’ life satisfaction, we use a simple regression model. Table 3 shows that the predictor (meaningful work) explains 28% of the variance of the outcome variable (R^2^ = 0.28, F (1.60) = 24.693, *p* < 0.00).

Table 4 shows that meaningful work is strongly related to life satisfaction in the research sample (*r* = 0.54, *p* < 0.00).

## 5. Discussion

Work is a fundamental area of human existence [7] and is worthy of the attention this topic receives in the literature. For people who experience marginalization or social disadvantages, having a meaningful job that that (a) provides a sense of growth and purpose and (b) generates satisfaction in their lives is a great challenge.

Although life satisfaction has been a topic of academic interest, this construct in the context of meaningful work has received little attention as an explanatory factor of personal well-being [54]. In addition to the above, the relationship between these two variables from perspectives that recognize the particularities of populations in conditions of marginalization and social disadvantage, such as the psychology of working theory (PWT) proposed by Duffy et al. [42], has been less than adequately studied. Consequently, we set out to analyze the effect that meaningful work has on general satisfaction with life in a group of people affected by the armed conflict in Colombia.

The results obtained in the study indicate that for the sample of the study, a relationship is observed between meaningful work and general satisfaction with life which is consistent with empirical evidence from another populations [6,53,54]. As stated by Allan et al. [13], when people develop their economic activity in a context that provides work full of meaning, they have the possibility of achieving personal fulfillment and self-development. Translating these approaches to the results of the study, it is possible to point out that the study participants experience a life full of purpose.

On the other hand, according to Duffi et al. [42] if work experiences promotes interpersonal connections and psychological adjustments, those conditions may contribute to increasing the satisfaction with life; indeed, the satisfaction of psychological needs is beneficial to improvements in well-being [42]. In our research, we believe that participants feel greater satisfaction with life because of the possibility to relate with other people and have a sense of belonging.

In addition to the abovementioned, it is reasonable to observe the existence of statistically significant relationships between meaningful work and well-being among people participating in this supported employment program, given that strategy helps people experiencing social disadvantages obtain paid employment and promotes social integration, people’s empowerment, and respect for human beings. Complementarily, having meaningful work helps to fulfill the needs of a population enduring marginalizing conditions, such as people involved in armed conflicts.

Finally, although organization studies have investigated meaningful work as a construct [65], authors have highlighted the necessity to continue delving into this topic [7,8,9] because the research on how relationship dynamics regarding work are associated with levels of general satisfaction with life among collaborators has been limited [66].

## 6. Conclusions

Several elements that merit mention in the conclusions have been identified in the research. First, we assert that providing meaningful work to marginalized populations can be an appropriate mechanism to promote their life satisfaction because this provision helps fulfill the need for social connection and self-determination.

By conducting this research, we contribute to closing the gap in the literature regarding how certain working conditions affect satisfaction with life, specifically, with people who have been marginalized. It has even been assumed that the population affected by the armed conflict that has employment is a population that shares similar characteristics with the general population; they have conditions of marginalization and social disadvantage because of their insufficient credentials, low educational levels, and insufficient skills to participate in the urban labor market, which could affect their experiences in the workplace.

In addition to the above, the results help correct the gaps in the literature identified by authors who have indicated that the reintegration process in fragile or post-conflict countries has been under-examined and that a more research on this process is required from the perspective of people in the reintegration process. The information obtained here contributes to developing knowledge in that direction.

The study of experiences at work, using populations such as the one that participated in this investigation is of great importance in affected conflict countries. For example, Colombia has more than nine million internally displaced people; the civilian population affected by the conflict exceeds 300,000, and more than 60,000 members of illegal armed groups have left them and are about to complete, or have completed, their reintegration process. Hence, continued exploration of this theoretical perspective is appropriate, given that the objective study group is large.

In addition to the above, these results are significant for international institutions, public policymakers, organizations, and professional counselors who want to effectively help people involved in armed conflicts. Sharing this approach with international institutions and governments managing wars or significant migrations could help them generate appropriate policies. According to the Organización de las Naciones Unidas [67], Venezuela’s migration has been one of the largest in the last years; thus, an analysis of the migrants’ work experiences in the host countries would be worthwhile.

On the other hand, the relevance of this study for policy makers and organizations consists of identifying good practices that are being carried out in companies to favor the labor inclusion of people affected by the armed conflict and to define or redefine tax incentives that encourage the business sector to participate in initiatives of this type. As mentioned above, this study may be useful for professional counselors, especially those practicing in Colombia. Given that the significant results are given in an employment program based on the principles of supported employment, it is conceivable that the practices adopted here could be applicable to other programs to be developed, especially since the post-conflict process in Colombia will last more than 10 years, according to some specialists.

This study has limitations. Considering that, in Colombia, the population in the reintegration process exceeds 60,000, and that, of the 7183 who are employed, 1723 work in the formal sector, it would be desirable to conduct a study with a statistically representative sample. The same applies to people who have been victims of the armed conflict, since 55% of this population is in working age. Additionally, the results of this study describe the experience of a group of people working in a specific city; therefore, conducting additional studies in other cities would be appropriate. In complement to the above, administering the questionnaires individually could be problematic because the participants may have felt fearful, imagining that their answers could be easily identified and, therefore, generate a social desirability effect.

Though the individuals in this research are participating in an employment program based on the supported employment model, which promotes social integration and the empowerment of individuals, it is not possible to form conclusions regarding this statistical relationship; therefore, it is necessary to carry out further research to study this possible link.

Regarding novel lines of research, the relationship between meaningful work and variables such as depression, anxiety, and exposure to risk factors could be studied and would be of special importance for people affected by armed conflicts because they endure psychosocial problems. Conducting longitudinal, qualitative, or mixed methods studies to more closely investigate these issues would also be worthwhile.

Additionally, studies could be conducted to analyze the relationship between meaningful work and variables of organizational and social levels to advance the understanding of this phenomenon.

## Figures and Tables

**Table 1:** Fit indexes of WAMI factorial structure.

Models	χ^2^ (p)	CMIN/DF	GFI	NFI	CFI	RMSEA
Three factors	197.797 (0.000)	197.797	0.677	0.735	0.756	0.345
One factor	23.300 (0.106)	1.456	0.919	0.969	0.990	0.086

Source: Authors.

**Table 2:** Means, standard deviations, and correlations.

Variables	M	SD	1	2
1. Meaningful work (Meaning)	4.8	0.445	1	0.54 **
2. Satisfaction with life (LifeSatis)	4.1	0.760	0.54 **	1

Note: ** *p* < 0.01 bilateral. Source: Authors.

**Table 3:** Regression Model.

Model	R	R Square	Adjusted R Square	Stand. Error of the Estimate	Change Statistics					Durbin–Watson
					R Square Change	Change in F	gl1	gl2	Sig. Change in F	
1	0.540 ^a^	0.292	0.280	0.84867216	0.292	24.693	1	60	0.000	2.068

^a^ Predictor variables: (Constant), Score Z (Meaningful).

**Table 4:** Coefficients Model.

	Nonstandard Coefficients		Standard Coefficients	T	Sig.
	B	Std. Error	Beta		
(Constant)	7.065 × 10^−16^	0.108		0.000	1.000
Score Z (LifeSatis)	0.540	0.109	0.540	4.969	0.000

Source: Authors.

## Data Availability

Available upon request.
